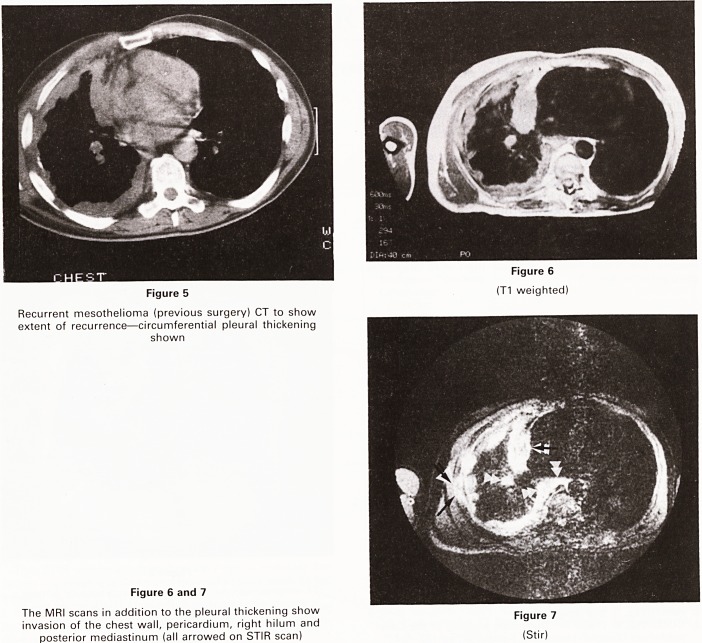# MRI in Thoracic Malignancy

**Published:** 1988-05

**Authors:** P Goddard


### MRI in Thoracic Malignancy

**P Goddard MD, FRCR**

Early reports established a role for MRI in the investigation of thoracic malignancy. Gamsu et al (1) showed two main advantages over CT in the mediastinum: ease in distinguishing between lymph nodes and vascular structures and very high contrast between lesions and fatty tissue.

The ability to scan in any desired plane is also an advantage. If direct comparison with chest X-rays is desired the coronal plane can be very useful (Figure 2). If comparison with CT is necessary, the transverse plane is indicated (Figures 6, 7, 10 and 11). The sagittal plane can be difficult to interpret in the chest but is the most useful plane for studying the spine.

Cardiac gating (2) enables clearer images to be obtained and cardiovascular abnormalities, such as aneurysms, to be distinguished from neoplasia. Ungated scans are, however, much quicker to perform than gated scans and can often produce useful results. It is therefore sensible to start scanning with a short sequence, such as T1 weighted coronal scans (taking between 6 and 10 minutes) and only proceed to gating if necessary. All the examples shown in this article are ungated. The use of several sequences may also assist in showing whether a mass is solid, cystic, fatty or haematoma.

MRI can be effectively used to confirm or refute the presence of large lymph nodes in the mediastinum and this can be particularly useful in patients with carcinoma of the bronchus (Figures 1–7).

Magnetic Resonance Imaging is also useful for studying the pleura and chest wall (3) (Figures 8–11).

MRI has an intrinsic disadvantage in the examination of the lung fields in that good images of the normal lung parenchyma are not routinely obtained due to the low proton density and hence low signal. This does mean, however, that solid lesions of high proton density stand out against the low signal background and that such lesions are more readily studied than was originally thought.

**Acknowledgements**
Acknowledgements are due to the radiographers and secretaries at the Bristol MRI Scanner Centre, Mr Alan Wood (engineer) and to my helpful colleagues, Dr G. Laszlo and Dr J Catterral.

**REFERENCES**

1. GAMSU, G. WEBB, W. R. SHELDON, P. KAUFMAN L. CROOKS, L. BIRNBERG, F. A. GOODMAN, P. HINCHLIFFE, W. A. HEDGECOCK, M. L. (1983) "Nuclear magnetic resonance imaging of the thorax" *Radiology* **147**, 473–480.
2. HARTNELL, G. this issue.
3. GODDARD, P. (1987) in "Diagnostic Imaging of the Chest" publishers Churchill Livinstone, Edinburgh, pp 123–124.

**Figure 1**

X-ray showing a large apical carcinoma of the bronchus

**Figure 2**

T1 weighted coronal MR scan. The large necrotic tumour is shown with extension to the aorta (black arrow) and a large lymph node at the left hilum (white arrow). The coronal plane is the most readily comparable with the PA chest radiograph

**Figure 3**

Patient with Ca broncus CT scans showed a possible lymph node in the sub-carinal region (black arrow). Confusion with the top of the left atrium was considered possible

**Figure 4**

MR scans (transverse) confirmed the sub-carinal node (black arrow) and showed a node at the right hilum (white arrows)

**Figure 5**

Recurrent mesothelioma (previous surgery) CT to show extent of recurrence—circumferential pleural thickening shown

**Figure 6**

(T1 weighted)

**Figure 7**

(Stir)

**Figure 6 and 7**

The MRI scans in addition to the pleural thickening show invasion of the chest wall, pericardium, right hilum and posterior mediastinum (all arrowed on STIR scan)

## Figures and Tables

**Figure 1 f1:**
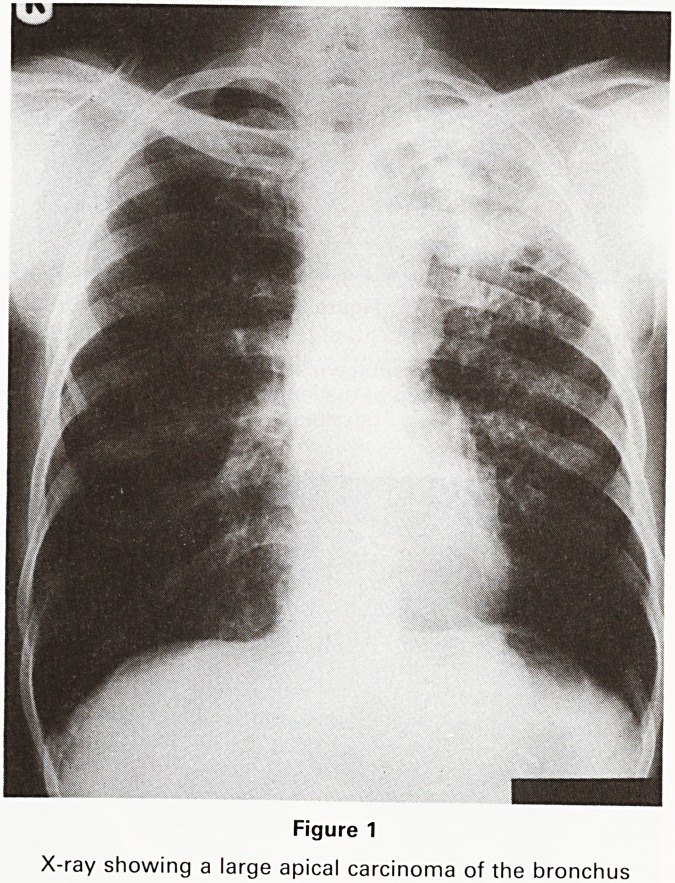


**Figure 2 f2:**
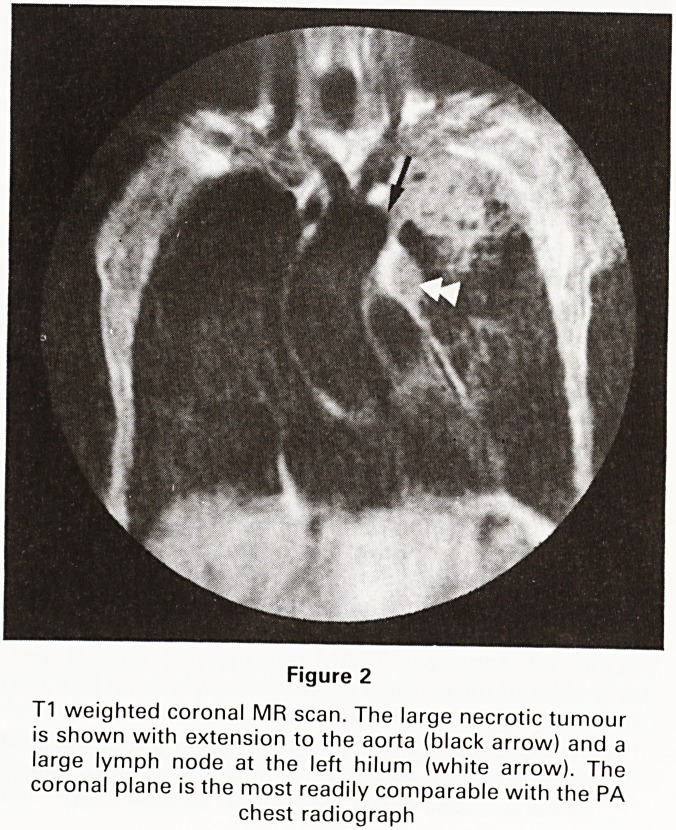


**Figure 3 f3:**
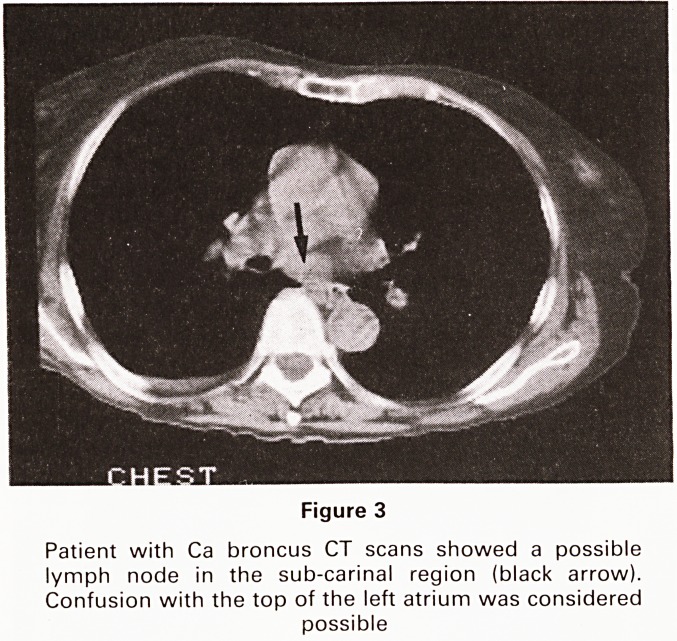


**Figure 4 f4:**
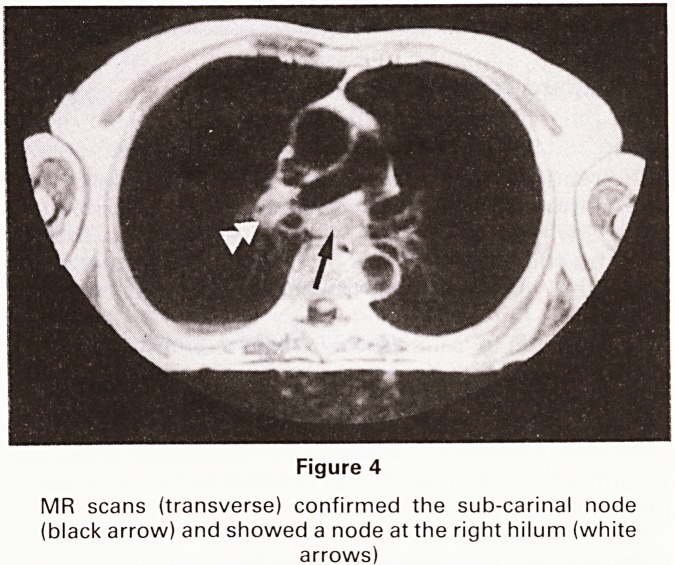


**Figure 5 Figure 6 Figure 7 f5:**